# Dinitro­sylbis[tris­(4-methyl­phen­yl)phosphane]iron

**DOI:** 10.1107/S160053681100465X

**Published:** 2011-02-12

**Authors:** Myron W. Jones, Douglas R. Powell, George B. Richter-Addo

**Affiliations:** a Department of Chemistry and Biochemistry, University of Oklahoma, 101 Stephenson Parkway, Norman, OK 73019-5251, USA

## Abstract

The title compound, [Fe(NO)_2_(C_21_H_21_P)_2_], belongs to the family of metal dinitrosyl compounds with the general formula Fe(NO)_2_(*L*)_*x*_, referred to collectively as ‘dinitrosyl iron compounds’ (DNICs). Herein we report the structure of a dinitrosyl iron diphosphane complex, [Fe(NO)_2_ *L*_2_], with *L* = P(C_6_H_4_-*p*-CH_3_)_3_. There are two crystallographically independent but chemically equal mol­ecules per asymmetric unit. The iron atom in each mol­ecule is tetra­hedrally coordinated by two phosphane ligands and two NO groups, with Fe—N—O angles in the range 173.84 (15)–179.31 (16)°.

## Related literature

The starting compound, Fe(NO)_2_(CO)_2_, was prepared using a published method described by Eisch & King (1965 ▶). For the structures of some related dinitrosyl complexes, see: Li *et al.* (2003 ▶); Atkinson *et al.* (1996 ▶); Li Kam Wah *et al.* (1989 ▶); Albano *et al.* (1974 ▶). For general information on metal nitrosyl chemistry, see: Richter-Addo & Legzdins (1992 ▶).
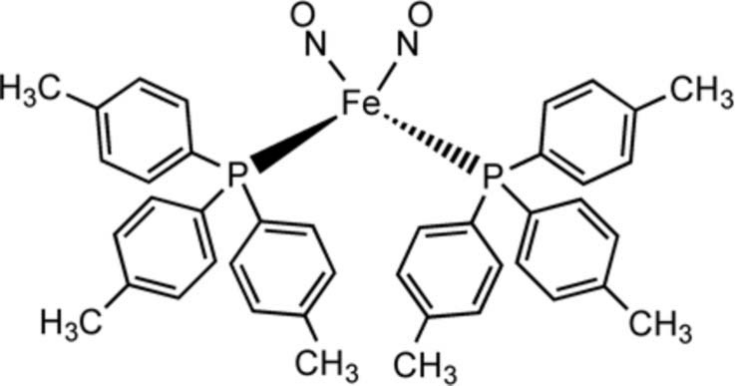


## Experimental

### 

#### Crystal data

[Fe(NO)_2_(C_21_H_21_P)_2_]
*M* *_r_* = 724.57
Triclinic, 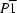

*a* = 10.240 (2) Å
*b* = 19.409 (4) Å
*c* = 21.040 (4) Å
α = 114.508 (5)°
β = 93.158 (6)°
γ = 94.732 (6)°
*V* = 3773.5 (13) Å^3^
*Z* = 4
Mo *K*α radiation
μ = 0.52 mm^−1^
*T* = 100 K
0.34 × 0.29 × 0.29 mm

#### Data collection

Bruker APEX CCD diffractometer
Absorption correction: multi-scan (*SADABS*; Sheldrick, 2001 ▶) *T*_min_ = 0.843, *T*_max_ = 0.863
39881 measured reflections
14767 independent reflections
12772 reflections with *I* > 2σ(*I*)
*R*_int_ = 0.025

#### Refinement

*R*[*F* ^2^ > 2σ(*F* ^2^)] = 0.035
*wR*(*F* ^2^) = 0.095
*S* = 1.01
14767 reflections
883 parameters
H-atom parameters constrained
Δρ_max_ = 0.47 e Å^−3^
Δρ_min_ = −0.26 e Å^−3^

### 

Data collection: *SMART* (Bruker, 2007 ▶); cell refinement: *SAINT* (Bruker, 2007 ▶); data reduction: *SAINT*; program(s) used to solve structure: *SHELXTL* (Sheldrick, 2008 ▶); program(s) used to refine structure: *SHELXTL*; molecular graphics: *SHELXTL*; software used to prepare material for publication: *SHELXTL*.

## Supplementary Material

Crystal structure: contains datablocks I, global. DOI: 10.1107/S160053681100465X/fk2034sup1.cif

Structure factors: contains datablocks I. DOI: 10.1107/S160053681100465X/fk2034Isup2.hkl

Additional supplementary materials:  crystallographic information; 3D view; checkCIF report

## Figures and Tables

**Fig. 1.**
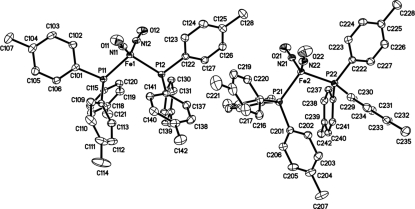
The molecular structure of the title compound showing the relative orientation of the two neighboring molecules. Hydrogen atoms were omitted for clarity. The displacement ellipsoids were drawn at the 50% probability level.

## supplementary crystallographic information

### Comment

The molecular structure of the title compound is shown in Fig. 1. There are two distinct molecules per asymmetric unit of the cell. Each molecule possesses a distorted tetrahedral geometry around the iron center. The irons are bound to two nitrosyl groups *via* the nitrogen atoms and to two phosphine ligands *via* the phosphorous atoms. The Fe(NO)_2_ groups are in the *attracto* conformation where the bond angles O···Fe···O < N—Fe—N (Richter-Addo & Legzdins, 1992). The N—Fe—N bond angles for molecule 1 and molecule 2 are 129.89 (7)° and 124.29 (8)° respectively, while the interphosphine angles, P—Fe—P, are 106.00 (2)° and 105.57 (2)° respectively. The Fe—N—O bond angles range between 173.84 (15)° and 179.31 (16)°. For the structures of some related complexes, see: Li *et al.* (2003), Atkinson *et al.* (1996), Li Kam Wah *et al.* (1989), and Albano *et al.* (1974).

### Experimental

Dark red Fe(NO)_2_(CO)_2_ (20 µ*L*, 0.18 mmol) (Eisch & King, 1965) was added by syringe under nitrogen to a colorless toluene solution (5 ml) of P(C_6_H_4_-*p*-CH_3_)_3_ (0.111 g, 0.36 mmol) in a Schlenk tube. The mixture was stirred and heated to reflux under nitrogen for a period of ~3 h. A color change from light red to black/dark brown was observed within the first 30 min. The reaction was stopped when the infrared spectrum indicated the absence of characteristic carbonyl stretching frequencies for Fe(NO)_2_(CO)_2_ (ν_CO_ = 2090 cm^-1^ and 2040 cm^-1^). The reaction mixture was filtered through celite under N_2_ and the solvent was subsequently removed under vacuum. Isolated yield of the Fe(NO)_2_*L*_2_ compound: 31%. IR (toluene, cm^-1^): ν_NO_ = 1714 s and 1670 s. ^31^P{^1^H} NMR (CDCl_3_): δ 58.4 (*s*) referenced to 85% H_3_PO_4_. Suitable crystals for X-ray diffraction studies were grown by slow evaporation of a chloroform solution of the complex under nitrogen at ambient temperature.

### Refinement

Phenyl H atoms were placed using known geometry with C—H = 0.95 Å. Methyl H atoms were initially located on a difference map and their positions were refined as rigid groups maintaining C—H = 0.98 Å and C—C—H angles to be equal by refining a torsion angle for each of the rigid groups. Once refinement had converged, the additional torsion angles were removed and refinement was repeated. Displacement parameters of phenyl H atoms were set to 1.2 times the isotropic equivalent for the bonded C (1.5 for methyl H atoms).

### Crystal data

**Table:**

[Fe(NO)_2_(C_21_H_21_P)_2_]	*Z* = 4
*M**_r_* = 724.57	*F*(000) = 1520
Triclinic, *P*1	*D*_x_ = 1.275 Mg m^−^^3^
Hall symbol: -P 1	Mo *K*α radiation, λ = 0.71073 Å
*a* = 10.240 (2) Å	Cell parameters from 8062 reflections
*b* = 19.409 (4) Å	θ = 2.3–28.3°
*c* = 21.040 (4) Å	µ = 0.52 mm^−^^1^
α = 114.508 (5)°	*T* = 100 K
β = 93.158 (6)°	Prism, red
γ = 94.732 (6)°	0.34 × 0.29 × 0.29 mm
*V* = 3773.5 (13) Å^3^	

### Data collection

**Table:**

Bruker APEX CCD diffractometer	14767 independent reflections
Radiation source: fine-focus sealed tube	12772 reflections with *I* > 2σ(*I*)
graphite	*R*_int_ = 0.025
ω scans	θ_max_ = 26.0°, θ_min_ = 1.9°
Absorption correction: multi-scan (*SADABS*; Sheldrick, 2001)	*h* = −12→12
*T*_min_ = 0.843, *T*_max_ = 0.863	*k* = −23→23
39881 measured reflections	*l* = −25→25

### Refinement

**Table:**

Refinement on *F*^2^	Primary atom site location: structure-invariant direct methods
Least-squares matrix: full	Secondary atom site location: difference Fourier map
*R*[*F*^2^ > 2σ(*F*^2^)] = 0.035	Hydrogen site location: geom and difmap
*wR*(*F*^2^) = 0.095	H-atom parameters constrained
*S* = 1.01	*w* = 1/[σ^2^(*F*_o_^2^) + (0.052*P*)^2^ + 1.6*P*] where *P* = (*F*_o_^2^ + 2*F*_c_^2^)/3
14767 reflections	(Δ/σ)_max_ = 0.002
883 parameters	Δρ_max_ = 0.47 e Å^−^^3^
0 restraints	Δρ_min_ = −0.26 e Å^−^^3^

### Fractional atomic coordinates and isotropic or equivalent isotropic displacement parameters (Å^2^)

**Table:**

	*x*	*y*	*z*	*U*_iso_*/*U*_eq_	
Fe1	0.39525 (2)	0.721825 (14)	0.379710 (13)	0.01684 (7)	
P11	0.28352 (4)	0.75740 (2)	0.30476 (2)	0.01665 (10)	
N11	0.52762 (15)	0.78538 (9)	0.40581 (8)	0.0229 (3)	
O11	0.62231 (14)	0.83142 (9)	0.42413 (9)	0.0423 (4)	
C101	0.38308 (17)	0.76140 (10)	0.23620 (9)	0.0187 (4)	
C102	0.50084 (18)	0.72935 (11)	0.22635 (10)	0.0243 (4)	
H102	0.5286	0.7048	0.2547	0.029*	
C103	0.57844 (19)	0.73288 (12)	0.17537 (10)	0.0296 (4)	
H103	0.6592	0.7112	0.1699	0.036*	
C104	0.54089 (18)	0.76711 (11)	0.13254 (10)	0.0259 (4)	
C105	0.42240 (19)	0.79844 (11)	0.14174 (10)	0.0272 (4)	
H105	0.3939	0.8216	0.1124	0.033*	
C106	0.34516 (18)	0.79640 (11)	0.19310 (10)	0.0239 (4)	
H106	0.2654	0.8191	0.1991	0.029*	
C107	0.6249 (2)	0.77143 (14)	0.07718 (11)	0.0379 (5)	
H10A	0.6896	0.8169	0.0982	0.057*	
H10B	0.5688	0.7744	0.0393	0.057*	
H10C	0.6709	0.7258	0.0581	0.057*	
C108	0.23570 (18)	0.85384 (10)	0.34316 (9)	0.0200 (4)	
C109	0.3295 (2)	0.91604 (10)	0.35781 (10)	0.0251 (4)	
H109	0.4153	0.9077	0.3434	0.030*	
C110	0.2992 (2)	0.98989 (11)	0.39310 (11)	0.0340 (5)	
H110	0.3646	1.0315	0.4027	0.041*	
C111	0.1744 (2)	1.00388 (12)	0.41462 (11)	0.0393 (6)	
C112	0.0815 (2)	0.94192 (13)	0.40057 (10)	0.0365 (5)	
H112	−0.0040	0.9505	0.4154	0.044*	
C113	0.11095 (19)	0.86772 (12)	0.36529 (10)	0.0269 (4)	
H113	0.0457	0.8262	0.3562	0.032*	
C114	0.1395 (3)	1.08414 (14)	0.45209 (15)	0.0675 (10)	
H10D	0.0458	1.0851	0.4407	0.101*	
H10E	0.1923	1.1181	0.4370	0.101*	
H10F	0.1579	1.1013	0.5029	0.101*	
C115	0.13121 (17)	0.69818 (10)	0.25660 (9)	0.0187 (4)	
C116	0.04992 (18)	0.71691 (11)	0.21226 (10)	0.0260 (4)	
H116	0.0735	0.7621	0.2063	0.031*	
C117	−0.06413 (18)	0.67082 (11)	0.17700 (10)	0.0267 (4)	
H117	−0.1170	0.6841	0.1462	0.032*	
C118	−0.10355 (18)	0.60487 (11)	0.18570 (10)	0.0250 (4)	
C119	−0.02243 (19)	0.58641 (10)	0.22993 (10)	0.0252 (4)	
H119	−0.0468	0.5417	0.2366	0.030*	
C120	0.09349 (18)	0.63199 (10)	0.26454 (9)	0.0214 (4)	
H120	0.1479	0.6178	0.2941	0.026*	
C121	−0.2297 (2)	0.55519 (12)	0.14826 (12)	0.0361 (5)	
H10G	−0.3037	0.5762	0.1748	0.054*	
H10H	−0.2242	0.5035	0.1445	0.054*	
H10I	−0.2432	0.5536	0.1012	0.054*	
P12	0.27399 (4)	0.74526 (2)	0.47036 (2)	0.01681 (10)	
N12	0.38322 (14)	0.62764 (9)	0.34965 (8)	0.0214 (3)	
O12	0.37303 (14)	0.56049 (7)	0.33303 (8)	0.0337 (3)	
C122	0.34119 (18)	0.70741 (10)	0.53047 (9)	0.0203 (4)	
C123	0.47703 (19)	0.71779 (12)	0.54674 (10)	0.0280 (4)	
H123	0.5321	0.7406	0.5242	0.034*	
C124	0.5331 (2)	0.69512 (13)	0.59559 (11)	0.0352 (5)	
H124	0.6258	0.7039	0.6069	0.042*	
C125	0.4554 (2)	0.65974 (13)	0.62823 (11)	0.0323 (5)	
C126	0.3199 (2)	0.64810 (11)	0.61068 (10)	0.0265 (4)	
H126	0.2651	0.6231	0.6316	0.032*	
C127	0.26342 (18)	0.67224 (10)	0.56331 (9)	0.0222 (4)	
H127	0.1704	0.6647	0.5531	0.027*	
C128	0.5146 (2)	0.63598 (17)	0.68217 (14)	0.0505 (7)	
H10J	0.4522	0.6402	0.7169	0.076*	
H10K	0.5962	0.6692	0.7057	0.076*	
H10L	0.5340	0.5831	0.6590	0.076*	
C129	0.10421 (17)	0.70029 (10)	0.44695 (9)	0.0185 (4)	
C130	0.07860 (18)	0.62120 (10)	0.42010 (9)	0.0212 (4)	
H130	0.1489	0.5918	0.4188	0.025*	
C131	−0.04789 (18)	0.58485 (11)	0.39528 (10)	0.0242 (4)	
H131	−0.0632	0.5309	0.3778	0.029*	
C132	−0.15330 (18)	0.62621 (11)	0.39555 (10)	0.0260 (4)	
C133	−0.12697 (18)	0.70518 (11)	0.42229 (10)	0.0262 (4)	
H133	−0.1971	0.7346	0.4232	0.031*	
C134	−0.00062 (18)	0.74192 (10)	0.44770 (9)	0.0218 (4)	
H134	0.0145	0.7959	0.4658	0.026*	
C135	−0.2907 (2)	0.58681 (12)	0.36654 (12)	0.0367 (5)	
H10M	−0.3544	0.6161	0.3968	0.055*	
H10N	−0.2975	0.5354	0.3649	0.055*	
H10O	−0.3094	0.5834	0.3191	0.055*	
C136	0.26292 (17)	0.84432 (10)	0.53071 (9)	0.0185 (4)	
C137	0.19553 (18)	0.86158 (11)	0.59036 (9)	0.0234 (4)	
H137	0.1518	0.8215	0.5988	0.028*	
C138	0.19201 (19)	0.93630 (11)	0.63711 (10)	0.0276 (4)	
H138	0.1456	0.9469	0.6774	0.033*	
C139	0.25514 (18)	0.99655 (11)	0.62653 (10)	0.0258 (4)	
C140	0.32166 (19)	0.97939 (11)	0.56705 (10)	0.0246 (4)	
H140	0.3648	1.0195	0.5585	0.029*	
C141	0.32592 (18)	0.90400 (10)	0.51970 (9)	0.0214 (4)	
H141	0.3725	0.8933	0.4794	0.026*	
C142	0.2541 (2)	1.07824 (12)	0.67967 (11)	0.0372 (5)	
H10P	0.1701	1.0837	0.7004	0.056*	
H10Q	0.2652	1.1124	0.6563	0.056*	
H10R	0.3265	1.0914	0.7167	0.056*	
Fe2	0.20249 (2)	0.726480 (14)	0.861160 (13)	0.01703 (7)	
P21	0.07337 (4)	0.78326 (2)	0.81153 (2)	0.01688 (10)	
N21	0.19663 (14)	0.63819 (9)	0.80051 (8)	0.0217 (3)	
O21	0.19345 (15)	0.57497 (8)	0.75630 (8)	0.0366 (4)	
C201	−0.05695 (17)	0.83832 (10)	0.85539 (9)	0.0196 (4)	
C202	−0.03062 (19)	0.88754 (10)	0.92638 (10)	0.0224 (4)	
H202	0.0550	0.8936	0.9492	0.027*	
C203	−0.1274 (2)	0.92766 (10)	0.96401 (10)	0.0267 (4)	
H203	−0.1075	0.9607	1.0124	0.032*	
C204	−0.25337 (19)	0.92016 (11)	0.93188 (11)	0.0294 (4)	
C205	−0.27994 (19)	0.87105 (11)	0.86129 (11)	0.0296 (4)	
H205	−0.3656	0.8651	0.8386	0.036*	
C206	−0.18345 (18)	0.83046 (11)	0.82327 (10)	0.0245 (4)	
H206	−0.2038	0.7971	0.7750	0.029*	
C207	−0.3572 (2)	0.96526 (12)	0.97353 (13)	0.0422 (6)	
H2OA	−0.3894	0.9424	1.0042	0.063*	
H20B	−0.4305	0.9646	0.9413	0.063*	
H20C	−0.3189	1.0180	1.0021	0.063*	
C208	−0.01124 (17)	0.71612 (10)	0.72718 (9)	0.0187 (4)	
C209	−0.07366 (18)	0.64768 (10)	0.72364 (10)	0.0230 (4)	
H209	−0.0690	0.6374	0.7641	0.028*	
C210	−0.14236 (18)	0.59459 (11)	0.66173 (10)	0.0261 (4)	
H210	−0.1848	0.5485	0.6604	0.031*	
C211	−0.15029 (18)	0.60753 (11)	0.60152 (10)	0.0264 (4)	
C212	−0.08842 (19)	0.67611 (11)	0.60524 (10)	0.0267 (4)	
H212	−0.0929	0.6863	0.5648	0.032*	
C213	−0.02019 (18)	0.72984 (11)	0.66754 (10)	0.0231 (4)	
H213	0.0207	0.7764	0.6692	0.028*	
C214	−0.2240 (2)	0.54858 (13)	0.53420 (11)	0.0407 (5)	
H20D	−0.2704	0.5742	0.5098	0.061*	
H20E	−0.2878	0.5155	0.5450	0.061*	
H20F	−0.1615	0.5178	0.5041	0.061*	
C215	0.18434 (17)	0.84965 (10)	0.79286 (9)	0.0199 (4)	
C216	0.1841 (2)	0.92808 (11)	0.82258 (10)	0.0267 (4)	
H216	0.1147	0.9503	0.8495	0.032*	
C217	0.2857 (2)	0.97461 (12)	0.81311 (11)	0.0341 (5)	
H217	0.2855	1.0284	0.8347	0.041*	
C218	0.3865 (2)	0.94394 (12)	0.77295 (11)	0.0329 (5)	
C219	0.38517 (19)	0.86522 (12)	0.74230 (10)	0.0286 (4)	
H219	0.4525	0.8430	0.7137	0.034*	
C220	0.28759 (18)	0.81904 (11)	0.75285 (10)	0.0245 (4)	
H220	0.2902	0.7654	0.7327	0.029*	
C221	0.4951 (2)	0.99342 (15)	0.76049 (14)	0.0492 (6)	
H20G	0.5807	0.9782	0.7694	0.074*	
H20H	0.4915	1.0470	0.7923	0.074*	
H20I	0.4834	0.9871	0.7118	0.074*	
P22	0.09066 (4)	0.71480 (3)	0.94568 (2)	0.01739 (10)	
N22	0.34007 (15)	0.78364 (9)	0.89869 (8)	0.0247 (3)	
O22	0.44468 (15)	0.81869 (9)	0.92377 (10)	0.0486 (4)	
C222	0.16869 (17)	0.65324 (10)	0.97918 (9)	0.0192 (4)	
C223	0.30279 (17)	0.64885 (10)	0.97346 (9)	0.0207 (4)	
H223	0.3474	0.6720	0.9476	0.025*	
C224	0.37193 (18)	0.61113 (10)	1.00497 (10)	0.0227 (4)	
H224	0.4634	0.6088	1.0004	0.027*	
C225	0.31008 (19)	0.57673 (10)	1.04306 (10)	0.0244 (4)	
C226	0.1750 (2)	0.57987 (12)	1.04768 (11)	0.0305 (5)	
H226	0.1303	0.5558	1.0728	0.037*	
C227	0.10521 (19)	0.61749 (11)	1.01627 (10)	0.0274 (4)	
H227	0.0134	0.6190	1.0200	0.033*	
C228	0.3873 (2)	0.53776 (12)	1.07865 (11)	0.0337 (5)	
H20J	0.3826	0.5632	1.1295	0.051*	
H20K	0.4794	0.5404	1.0686	0.051*	
H20L	0.3501	0.4843	1.0611	0.051*	
C229	0.08260 (17)	0.79809 (10)	1.02850 (9)	0.0197 (4)	
C230	0.17595 (18)	0.86123 (10)	1.04769 (10)	0.0224 (4)	
H230	0.2380	0.8623	1.0161	0.027*	
C231	0.17944 (19)	0.92279 (11)	1.11262 (10)	0.0256 (4)	
H231	0.2442	0.9654	1.1248	0.031*	
C232	0.09016 (19)	0.92330 (11)	1.16006 (10)	0.0262 (4)	
C233	−0.0038 (2)	0.86033 (12)	1.14066 (10)	0.0282 (4)	
H233	−0.0663	0.8597	1.1722	0.034*	
C234	−0.00769 (19)	0.79843 (11)	1.07609 (10)	0.0247 (4)	
H234	−0.0723	0.7559	1.0641	0.030*	
C235	0.0974 (2)	0.98930 (13)	1.23171 (11)	0.0366 (5)	
H20M	0.0089	0.9947	1.2474	0.055*	
H20N	0.1329	1.0363	1.2289	0.055*	
H20O	0.1549	0.9796	1.2651	0.055*	
C236	−0.07859 (17)	0.67165 (10)	0.91595 (9)	0.0189 (4)	
C237	−0.10360 (18)	0.59358 (10)	0.87334 (10)	0.0221 (4)	
H237	−0.0339	0.5626	0.8666	0.027*	
C238	−0.22833 (18)	0.56068 (11)	0.84085 (10)	0.0231 (4)	
H238	−0.2427	0.5075	0.8116	0.028*	
C239	−0.33384 (17)	0.60460 (11)	0.85051 (9)	0.0217 (4)	
C240	−0.30823 (18)	0.68238 (11)	0.89178 (10)	0.0227 (4)	
H240	−0.3779	0.7134	0.8982	0.027*	
C241	−0.18260 (17)	0.71601 (10)	0.92405 (9)	0.0211 (4)	
H241	−0.1675	0.7695	0.9518	0.025*	
C242	−0.47141 (18)	0.56809 (12)	0.81872 (10)	0.0277 (4)	
H20P	−0.5091	0.5424	0.8461	0.042*	
H20Q	−0.4685	0.5308	0.7702	0.042*	
H20R	−0.5261	0.6074	0.8192	0.042*	

### Atomic displacement parameters (Å^2^)

**Table:**

	*U*^11^	*U*^22^	*U*^33^	*U*^12^	*U*^13^	*U*^23^
Fe1	0.01472 (13)	0.01709 (13)	0.01718 (13)	0.00358 (10)	0.00093 (9)	0.00549 (10)
P11	0.0145 (2)	0.0170 (2)	0.0172 (2)	0.00310 (17)	0.00163 (17)	0.00579 (18)
N11	0.0184 (8)	0.0240 (8)	0.0253 (8)	0.0039 (6)	−0.0006 (6)	0.0094 (7)
O11	0.0236 (8)	0.0363 (9)	0.0614 (11)	−0.0091 (7)	−0.0071 (7)	0.0188 (8)
C101	0.0159 (8)	0.0178 (9)	0.0177 (8)	0.0002 (7)	0.0020 (7)	0.0030 (7)
C102	0.0231 (10)	0.0258 (10)	0.0245 (10)	0.0072 (8)	0.0043 (8)	0.0100 (8)
C103	0.0206 (10)	0.0356 (11)	0.0297 (11)	0.0088 (8)	0.0081 (8)	0.0091 (9)
C104	0.0221 (10)	0.0282 (10)	0.0197 (9)	−0.0035 (8)	0.0035 (7)	0.0036 (8)
C105	0.0248 (10)	0.0333 (11)	0.0235 (10)	0.0012 (8)	0.0026 (8)	0.0123 (8)
C106	0.0193 (9)	0.0298 (10)	0.0235 (9)	0.0059 (8)	0.0041 (7)	0.0113 (8)
C107	0.0297 (11)	0.0493 (14)	0.0320 (11)	0.0007 (10)	0.0116 (9)	0.0142 (10)
C108	0.0239 (9)	0.0213 (9)	0.0142 (8)	0.0079 (7)	0.0008 (7)	0.0060 (7)
C109	0.0281 (10)	0.0224 (10)	0.0231 (9)	0.0049 (8)	−0.0005 (8)	0.0079 (8)
C110	0.0476 (13)	0.0210 (10)	0.0280 (11)	0.0041 (9)	−0.0114 (9)	0.0067 (8)
C111	0.0509 (14)	0.0289 (11)	0.0260 (11)	0.0215 (10)	−0.0137 (10)	−0.0013 (9)
C112	0.0356 (12)	0.0434 (13)	0.0231 (10)	0.0248 (10)	0.0002 (9)	0.0033 (9)
C113	0.0255 (10)	0.0318 (11)	0.0218 (9)	0.0103 (8)	0.0018 (8)	0.0085 (8)
C114	0.076 (2)	0.0357 (14)	0.0576 (17)	0.0335 (14)	−0.0298 (15)	−0.0138 (12)
C115	0.0154 (8)	0.0198 (9)	0.0180 (8)	0.0030 (7)	0.0031 (7)	0.0048 (7)
C116	0.0215 (10)	0.0324 (11)	0.0285 (10)	0.0000 (8)	−0.0004 (8)	0.0181 (9)
C117	0.0184 (9)	0.0369 (11)	0.0257 (10)	0.0035 (8)	−0.0026 (8)	0.0147 (9)
C118	0.0186 (9)	0.0241 (10)	0.0232 (9)	0.0020 (7)	0.0001 (7)	0.0013 (8)
C119	0.0266 (10)	0.0172 (9)	0.0268 (10)	0.0011 (7)	0.0010 (8)	0.0049 (8)
C120	0.0214 (9)	0.0201 (9)	0.0200 (9)	0.0042 (7)	−0.0003 (7)	0.0057 (7)
C121	0.0278 (11)	0.0294 (11)	0.0399 (12)	−0.0030 (9)	−0.0094 (9)	0.0062 (9)
P12	0.0159 (2)	0.0166 (2)	0.0169 (2)	0.00352 (17)	0.00099 (17)	0.00579 (18)
N12	0.0178 (8)	0.0222 (8)	0.0214 (8)	0.0061 (6)	0.0023 (6)	0.0056 (6)
O12	0.0325 (8)	0.0175 (7)	0.0456 (9)	0.0052 (6)	0.0036 (7)	0.0075 (6)
C122	0.0228 (9)	0.0187 (9)	0.0191 (9)	0.0061 (7)	0.0019 (7)	0.0068 (7)
C123	0.0219 (10)	0.0391 (12)	0.0298 (10)	0.0061 (8)	0.0038 (8)	0.0206 (9)
C124	0.0230 (10)	0.0535 (14)	0.0369 (12)	0.0113 (10)	0.0022 (9)	0.0257 (11)
C125	0.0337 (11)	0.0419 (12)	0.0284 (11)	0.0155 (9)	0.0040 (9)	0.0197 (10)
C126	0.0311 (11)	0.0267 (10)	0.0247 (10)	0.0067 (8)	0.0069 (8)	0.0128 (8)
C127	0.0222 (9)	0.0225 (9)	0.0205 (9)	0.0040 (7)	0.0021 (7)	0.0073 (8)
C128	0.0413 (14)	0.083 (2)	0.0514 (15)	0.0225 (13)	0.0079 (11)	0.0486 (15)
C129	0.0169 (9)	0.0206 (9)	0.0172 (8)	0.0032 (7)	0.0028 (7)	0.0068 (7)
C130	0.0191 (9)	0.0218 (9)	0.0229 (9)	0.0069 (7)	0.0026 (7)	0.0087 (8)
C131	0.0239 (10)	0.0200 (9)	0.0239 (9)	0.0014 (7)	0.0014 (8)	0.0046 (8)
C132	0.0192 (9)	0.0279 (10)	0.0244 (10)	0.0031 (8)	0.0003 (7)	0.0049 (8)
C133	0.0182 (9)	0.0287 (10)	0.0282 (10)	0.0089 (8)	−0.0002 (8)	0.0076 (8)
C134	0.0232 (9)	0.0190 (9)	0.0207 (9)	0.0058 (7)	0.0016 (7)	0.0054 (7)
C135	0.0212 (10)	0.0334 (12)	0.0433 (13)	0.0006 (9)	−0.0037 (9)	0.0054 (10)
C136	0.0164 (8)	0.0191 (9)	0.0173 (8)	0.0046 (7)	−0.0013 (7)	0.0049 (7)
C137	0.0231 (10)	0.0237 (10)	0.0213 (9)	0.0024 (7)	0.0023 (7)	0.0075 (8)
C138	0.0255 (10)	0.0309 (11)	0.0194 (9)	0.0043 (8)	0.0042 (8)	0.0031 (8)
C139	0.0229 (10)	0.0233 (10)	0.0227 (9)	0.0048 (8)	−0.0031 (8)	0.0017 (8)
C140	0.0258 (10)	0.0203 (9)	0.0246 (10)	0.0002 (7)	−0.0030 (8)	0.0076 (8)
C141	0.0225 (9)	0.0219 (9)	0.0170 (9)	0.0035 (7)	0.0005 (7)	0.0054 (7)
C142	0.0373 (12)	0.0256 (11)	0.0342 (12)	0.0048 (9)	0.0022 (9)	−0.0019 (9)
Fe2	0.01515 (13)	0.01775 (13)	0.01839 (13)	0.00327 (10)	0.00339 (10)	0.00735 (10)
P21	0.0166 (2)	0.0161 (2)	0.0176 (2)	0.00260 (17)	0.00314 (17)	0.00653 (18)
N21	0.0187 (8)	0.0225 (8)	0.0254 (8)	0.0051 (6)	0.0041 (6)	0.0108 (7)
O21	0.0392 (9)	0.0199 (7)	0.0389 (8)	0.0069 (6)	0.0010 (7)	0.0004 (6)
C201	0.0201 (9)	0.0175 (9)	0.0241 (9)	0.0033 (7)	0.0065 (7)	0.0107 (7)
C202	0.0248 (10)	0.0186 (9)	0.0242 (9)	0.0035 (7)	0.0036 (7)	0.0092 (8)
C203	0.0320 (11)	0.0187 (9)	0.0275 (10)	0.0030 (8)	0.0101 (8)	0.0071 (8)
C204	0.0262 (10)	0.0190 (9)	0.0419 (12)	0.0051 (8)	0.0146 (9)	0.0099 (9)
C205	0.0187 (10)	0.0265 (10)	0.0430 (12)	0.0047 (8)	0.0056 (8)	0.0132 (9)
C206	0.0221 (10)	0.0235 (10)	0.0270 (10)	0.0039 (8)	0.0035 (8)	0.0095 (8)
C207	0.0299 (12)	0.0288 (11)	0.0598 (15)	0.0077 (9)	0.0216 (11)	0.0079 (11)
C208	0.0154 (8)	0.0184 (9)	0.0197 (9)	0.0036 (7)	0.0029 (7)	0.0049 (7)
C209	0.0223 (9)	0.0224 (9)	0.0246 (9)	0.0044 (7)	0.0017 (7)	0.0099 (8)
C210	0.0232 (10)	0.0199 (9)	0.0322 (10)	0.0019 (7)	0.0008 (8)	0.0084 (8)
C211	0.0217 (10)	0.0269 (10)	0.0238 (10)	0.0029 (8)	0.0004 (8)	0.0043 (8)
C212	0.0255 (10)	0.0332 (11)	0.0206 (9)	0.0022 (8)	0.0025 (8)	0.0108 (8)
C213	0.0214 (9)	0.0231 (9)	0.0250 (9)	0.0004 (7)	0.0038 (7)	0.0104 (8)
C214	0.0450 (14)	0.0353 (12)	0.0289 (11)	−0.0050 (10)	−0.0072 (10)	0.0041 (10)
C215	0.0198 (9)	0.0215 (9)	0.0189 (9)	−0.0006 (7)	0.0001 (7)	0.0097 (7)
C216	0.0342 (11)	0.0234 (10)	0.0218 (9)	0.0031 (8)	0.0036 (8)	0.0087 (8)
C217	0.0476 (13)	0.0205 (10)	0.0307 (11)	−0.0071 (9)	−0.0012 (10)	0.0098 (9)
C218	0.0303 (11)	0.0382 (12)	0.0308 (11)	−0.0124 (9)	−0.0062 (9)	0.0192 (10)
C219	0.0219 (10)	0.0405 (12)	0.0275 (10)	0.0012 (8)	0.0022 (8)	0.0190 (9)
C220	0.0244 (10)	0.0249 (10)	0.0253 (10)	0.0033 (8)	0.0035 (8)	0.0114 (8)
C221	0.0409 (14)	0.0538 (15)	0.0561 (16)	−0.0189 (12)	−0.0036 (12)	0.0317 (13)
P22	0.0158 (2)	0.0186 (2)	0.0188 (2)	0.00351 (17)	0.00334 (17)	0.00850 (18)
N22	0.0202 (8)	0.0242 (8)	0.0302 (9)	0.0030 (7)	0.0046 (7)	0.0117 (7)
O22	0.0238 (8)	0.0433 (10)	0.0653 (11)	−0.0094 (7)	0.0005 (8)	0.0124 (9)
C222	0.0207 (9)	0.0175 (9)	0.0186 (9)	0.0038 (7)	0.0016 (7)	0.0065 (7)
C223	0.0204 (9)	0.0183 (9)	0.0228 (9)	0.0016 (7)	0.0025 (7)	0.0080 (7)
C224	0.0185 (9)	0.0212 (9)	0.0253 (9)	0.0026 (7)	−0.0001 (7)	0.0070 (8)
C225	0.0285 (10)	0.0213 (9)	0.0232 (9)	0.0041 (8)	−0.0006 (8)	0.0092 (8)
C226	0.0328 (11)	0.0348 (11)	0.0355 (11)	0.0083 (9)	0.0095 (9)	0.0248 (10)
C227	0.0217 (10)	0.0325 (11)	0.0344 (11)	0.0063 (8)	0.0073 (8)	0.0193 (9)
C228	0.0348 (12)	0.0356 (12)	0.0376 (12)	0.0076 (9)	0.0021 (9)	0.0218 (10)
C229	0.0197 (9)	0.0225 (9)	0.0190 (9)	0.0075 (7)	0.0021 (7)	0.0099 (7)
C230	0.0210 (9)	0.0253 (10)	0.0224 (9)	0.0051 (7)	0.0027 (7)	0.0109 (8)
C231	0.0241 (10)	0.0254 (10)	0.0245 (10)	0.0020 (8)	−0.0040 (8)	0.0089 (8)
C232	0.0287 (10)	0.0299 (10)	0.0189 (9)	0.0123 (8)	0.0002 (8)	0.0078 (8)
C233	0.0292 (11)	0.0362 (11)	0.0226 (10)	0.0119 (9)	0.0083 (8)	0.0137 (9)
C234	0.0250 (10)	0.0273 (10)	0.0236 (9)	0.0054 (8)	0.0048 (8)	0.0118 (8)
C235	0.0402 (13)	0.0377 (12)	0.0244 (10)	0.0127 (10)	0.0035 (9)	0.0042 (9)
C236	0.0170 (9)	0.0235 (9)	0.0195 (9)	0.0028 (7)	0.0045 (7)	0.0118 (7)
C237	0.0183 (9)	0.0241 (9)	0.0263 (10)	0.0058 (7)	0.0054 (7)	0.0120 (8)
C238	0.0230 (9)	0.0218 (9)	0.0239 (9)	0.0003 (7)	0.0034 (7)	0.0092 (8)
C239	0.0185 (9)	0.0303 (10)	0.0198 (9)	0.0020 (7)	0.0024 (7)	0.0141 (8)
C240	0.0186 (9)	0.0300 (10)	0.0250 (9)	0.0073 (7)	0.0056 (7)	0.0160 (8)
C241	0.0217 (9)	0.0221 (9)	0.0217 (9)	0.0041 (7)	0.0060 (7)	0.0105 (8)
C242	0.0206 (10)	0.0366 (11)	0.0271 (10)	0.0009 (8)	−0.0002 (8)	0.0154 (9)

### Geometric parameters (Å, °)

**Table:**

Fe1—N11	1.6555 (16)	Fe2—N21	1.6519 (16)
Fe1—N12	1.6590 (16)	Fe2—N22	1.6529 (16)
Fe1—P12	2.2407 (6)	Fe2—P22	2.2451 (6)
Fe1—P11	2.2600 (6)	Fe2—P21	2.2574 (6)
P11—C108	1.8266 (18)	P21—C208	1.8232 (18)
P11—C115	1.8288 (18)	P21—C201	1.8242 (18)
P11—C101	1.8352 (18)	P21—C215	1.8259 (18)
N11—O11	1.191 (2)	N21—O21	1.190 (2)
C101—C102	1.389 (3)	C201—C206	1.394 (3)
C101—C106	1.397 (3)	C201—C202	1.395 (3)
C102—C103	1.390 (3)	C202—C203	1.385 (3)
C102—H102	0.9500	C202—H202	0.9500
C103—C104	1.379 (3)	C203—C204	1.390 (3)
C103—H103	0.9500	C203—H203	0.9500
C104—C105	1.389 (3)	C204—C205	1.388 (3)
C104—C107	1.511 (3)	C204—C207	1.511 (3)
C105—C106	1.385 (3)	C205—C206	1.389 (3)
C105—H105	0.9500	C205—H205	0.9500
C106—H106	0.9500	C206—H206	0.9500
C107—H10A	0.9800	C207—H2OA	0.9799
C107—H10B	0.9798	C207—H20B	0.9800
C107—H10C	0.9801	C207—H20C	0.9800
C108—C113	1.393 (3)	C208—C213	1.386 (3)
C108—C109	1.394 (3)	C208—C209	1.397 (3)
C109—C110	1.385 (3)	C209—C210	1.385 (3)
C109—H109	0.9500	C209—H209	0.9500
C110—C111	1.390 (3)	C210—C211	1.390 (3)
C110—H110	0.9500	C210—H210	0.9500
C111—C112	1.387 (3)	C211—C212	1.396 (3)
C111—C114	1.510 (3)	C211—C214	1.510 (3)
C112—C113	1.388 (3)	C212—C213	1.392 (3)
C112—H112	0.9500	C212—H212	0.9500
C113—H113	0.9500	C213—H213	0.9500
C114—H10D	0.9800	C214—H20D	0.9798
C114—H10E	0.9800	C214—H20E	0.9801
C114—H10F	0.9798	C214—H20F	0.9800
C115—C120	1.390 (3)	C215—C216	1.385 (3)
C115—C116	1.393 (3)	C215—C220	1.402 (3)
C116—C117	1.377 (3)	C216—C217	1.398 (3)
C116—H116	0.9500	C216—H216	0.9500
C117—C118	1.397 (3)	C217—C218	1.384 (3)
C117—H117	0.9500	C217—H217	0.9500
C118—C119	1.387 (3)	C218—C219	1.389 (3)
C118—C121	1.507 (3)	C218—C221	1.514 (3)
C119—C120	1.385 (3)	C219—C220	1.376 (3)
C119—H119	0.9500	C219—H219	0.9500
C120—H120	0.9500	C220—H220	0.9500
C121—H10G	0.9800	C221—H20G	0.9801
C121—H10H	0.9799	C221—H20H	0.9800
C121—H10I	0.9800	C221—H20I	0.9802
P12—C129	1.8237 (18)	P22—C236	1.8174 (18)
P12—C136	1.8298 (18)	P22—C229	1.8323 (18)
P12—C122	1.8361 (18)	P22—C222	1.8323 (18)
N12—O12	1.195 (2)	N22—O22	1.187 (2)
C122—C123	1.392 (3)	C222—C223	1.392 (3)
C122—C127	1.393 (3)	C222—C227	1.394 (3)
C123—C124	1.390 (3)	C223—C224	1.383 (3)
C123—H123	0.9500	C223—H223	0.9500
C124—C125	1.392 (3)	C224—C225	1.385 (3)
C124—H124	0.9500	C224—H224	0.9500
C125—C126	1.391 (3)	C225—C226	1.397 (3)
C125—C128	1.508 (3)	C225—C228	1.504 (3)
C126—C127	1.384 (3)	C226—C227	1.384 (3)
C126—H126	0.9500	C226—H226	0.9500
C127—H127	0.9500	C227—H227	0.9500
C128—H10J	0.9801	C228—H20J	0.9801
C128—H10K	0.9799	C228—H20K	0.9800
C128—H10L	0.9800	C228—H20L	0.9800
C129—C134	1.393 (2)	C229—C230	1.390 (3)
C129—C130	1.394 (2)	C229—C234	1.398 (3)
C130—C131	1.385 (3)	C230—C231	1.389 (3)
C130—H130	0.9500	C230—H230	0.9500
C131—C132	1.396 (3)	C231—C232	1.388 (3)
C131—H131	0.9500	C231—H231	0.9500
C132—C133	1.392 (3)	C232—C233	1.391 (3)
C132—C135	1.509 (3)	C232—C235	1.513 (3)
C133—C134	1.387 (3)	C233—C234	1.386 (3)
C133—H133	0.9500	C233—H233	0.9500
C134—H134	0.9500	C234—H234	0.9500
C135—H10M	0.9800	C235—H20M	0.9800
C135—H10N	0.9800	C235—H20N	0.9800
C135—H10O	0.9798	C235—H20O	0.9799
C136—C141	1.388 (3)	C236—C237	1.396 (3)
C136—C137	1.396 (3)	C236—C241	1.398 (2)
C137—C138	1.379 (3)	C237—C238	1.383 (3)
C137—H137	0.9500	C237—H237	0.9500
C138—C139	1.394 (3)	C238—C239	1.403 (3)
C138—H138	0.9500	C238—H238	0.9500
C139—C140	1.388 (3)	C239—C240	1.387 (3)
C139—C142	1.516 (3)	C239—C242	1.507 (3)
C140—C141	1.393 (3)	C240—C241	1.393 (3)
C140—H140	0.9500	C240—H240	0.9500
C141—H141	0.9500	C241—H241	0.9500
C142—H10P	0.9800	C242—H20P	0.9800
C142—H10Q	0.9800	C242—H20Q	0.9800
C142—H10R	0.9800	C242—H20R	0.9799
			
N11—Fe1—N12	129.89 (7)	N21—Fe2—N22	124.29 (8)
N11—Fe1—P12	108.61 (6)	N21—Fe2—P22	104.14 (6)
N12—Fe1—P12	98.23 (5)	N22—Fe2—P22	108.24 (6)
N11—Fe1—P11	101.15 (6)	N21—Fe2—P21	104.17 (6)
N12—Fe1—P11	111.19 (5)	N22—Fe2—P21	108.98 (6)
P12—Fe1—P11	106.00 (2)	P22—Fe2—P21	105.57 (2)
C108—P11—C115	103.82 (8)	C208—P21—C201	102.89 (8)
C108—P11—C101	101.42 (8)	C208—P21—C215	106.11 (8)
C115—P11—C101	104.38 (8)	C201—P21—C215	105.07 (8)
C108—P11—Fe1	115.61 (6)	C208—P21—Fe2	112.38 (6)
C115—P11—Fe1	117.49 (6)	C201—P21—Fe2	123.08 (6)
C101—P11—Fe1	112.28 (6)	C215—P21—Fe2	106.05 (6)
O11—N11—Fe1	179.31 (16)	O21—N21—Fe2	179.10 (16)
C102—C101—C106	117.99 (16)	C206—C201—C202	118.24 (17)
C102—C101—P11	119.80 (14)	C206—C201—P21	123.77 (14)
C106—C101—P11	122.21 (13)	C202—C201—P21	117.86 (14)
C101—C102—C103	120.56 (18)	C203—C202—C201	120.95 (18)
C101—C102—H102	119.7	C203—C202—H202	119.5
C103—C102—H102	119.7	C201—C202—H202	119.5
C104—C103—C102	121.55 (18)	C202—C203—C204	120.81 (18)
C104—C103—H103	119.2	C202—C203—H203	119.6
C102—C103—H103	119.2	C204—C203—H203	119.6
C103—C104—C105	118.00 (17)	C205—C204—C203	118.35 (18)
C103—C104—C107	121.88 (19)	C205—C204—C207	121.6 (2)
C105—C104—C107	120.12 (19)	C203—C204—C207	120.06 (19)
C106—C105—C104	121.03 (18)	C204—C205—C206	121.15 (19)
C106—C105—H105	119.5	C204—C205—H205	119.4
C104—C105—H105	119.5	C206—C205—H205	119.4
C105—C106—C101	120.86 (17)	C205—C206—C201	120.49 (18)
C105—C106—H106	119.6	C205—C206—H206	119.8
C101—C106—H106	119.6	C201—C206—H206	119.8
C104—C107—H10A	109.5	C204—C207—H2OA	109.5
C104—C107—H10B	109.5	C204—C207—H20B	109.5
H10A—C107—H10B	109.5	H2OA—C207—H20B	109.5
C104—C107—H10C	109.5	C204—C207—H20C	109.5
H10A—C107—H10C	109.5	H2OA—C207—H20C	109.5
H10B—C107—H10C	109.5	H20B—C207—H20C	109.5
C113—C108—C109	118.39 (17)	C213—C208—C209	118.54 (16)
C113—C108—P11	121.57 (15)	C213—C208—P21	124.11 (14)
C109—C108—P11	119.68 (14)	C209—C208—P21	117.32 (14)
C110—C109—C108	120.86 (19)	C210—C209—C208	120.63 (18)
C110—C109—H109	119.6	C210—C209—H209	119.7
C108—C109—H109	119.6	C208—C209—H209	119.7
C109—C110—C111	120.8 (2)	C209—C210—C211	121.13 (18)
C109—C110—H110	119.6	C209—C210—H210	119.4
C111—C110—H110	119.6	C211—C210—H210	119.4
C112—C111—C110	118.27 (19)	C210—C211—C212	118.17 (17)
C112—C111—C114	120.4 (2)	C210—C211—C214	120.45 (18)
C110—C111—C114	121.4 (2)	C212—C211—C214	121.38 (18)
C111—C112—C113	121.3 (2)	C213—C212—C211	120.82 (18)
C111—C112—H112	119.4	C213—C212—H212	119.6
C113—C112—H112	119.4	C211—C212—H212	119.6
C112—C113—C108	120.4 (2)	C208—C213—C212	120.70 (17)
C112—C113—H113	119.8	C208—C213—H213	119.7
C108—C113—H113	119.8	C212—C213—H213	119.7
C111—C114—H10D	109.5	C211—C214—H20D	109.5
C111—C114—H10E	109.5	C211—C214—H20E	109.5
H10D—C114—H10E	109.5	H20D—C214—H20E	109.5
C111—C114—H10F	109.5	C211—C214—H20F	109.5
H10D—C114—H10F	109.5	H20D—C214—H20F	109.5
H10E—C114—H10F	109.5	H20E—C214—H20F	109.5
C120—C115—C116	118.13 (16)	C216—C215—C220	118.19 (17)
C120—C115—P11	119.21 (13)	C216—C215—P21	124.84 (14)
C116—C115—P11	122.66 (14)	C220—C215—P21	116.40 (14)
C117—C116—C115	120.86 (18)	C215—C216—C217	120.13 (19)
C117—C116—H116	119.6	C215—C216—H216	119.9
C115—C116—H116	119.6	C217—C216—H216	119.9
C116—C117—C118	121.21 (18)	C218—C217—C216	121.34 (19)
C116—C117—H117	119.4	C218—C217—H217	119.3
C118—C117—H117	119.4	C216—C217—H217	119.3
C119—C118—C117	117.77 (17)	C217—C218—C219	118.31 (18)
C119—C118—C121	120.96 (18)	C217—C218—C221	122.0 (2)
C117—C118—C121	121.28 (18)	C219—C218—C221	119.7 (2)
C120—C119—C118	121.14 (18)	C220—C219—C218	120.81 (19)
C120—C119—H119	119.4	C220—C219—H219	119.6
C118—C119—H119	119.4	C218—C219—H219	119.6
C119—C120—C115	120.88 (17)	C219—C220—C215	121.18 (18)
C119—C120—H120	119.6	C219—C220—H220	119.4
C115—C120—H120	119.6	C215—C220—H220	119.4
C118—C121—H10G	109.5	C218—C221—H20G	109.5
C118—C121—H10H	109.5	C218—C221—H20H	109.5
H10G—C121—H10H	109.5	H20G—C221—H20H	109.5
C118—C121—H10I	109.5	C218—C221—H20I	109.5
H10G—C121—H10I	109.5	H20G—C221—H20I	109.5
H10H—C121—H10I	109.5	H20H—C221—H20I	109.5
C129—P12—C136	105.07 (8)	C236—P22—C229	105.45 (8)
C129—P12—C122	103.58 (8)	C236—P22—C222	105.88 (8)
C136—P12—C122	101.37 (8)	C229—P22—C222	99.71 (8)
C129—P12—Fe1	115.06 (6)	C236—P22—Fe2	113.70 (6)
C136—P12—Fe1	118.82 (6)	C229—P22—Fe2	120.17 (6)
C122—P12—Fe1	111.08 (6)	C222—P22—Fe2	110.27 (6)
O12—N12—Fe1	175.05 (15)	O22—N22—Fe2	173.84 (15)
C123—C122—C127	118.20 (17)	C223—C222—C227	118.43 (17)
C123—C122—P12	118.11 (14)	C223—C222—P22	117.38 (13)
C127—C122—P12	123.62 (14)	C227—C222—P22	123.83 (14)
C124—C123—C122	120.77 (18)	C224—C223—C222	120.75 (17)
C124—C123—H123	119.6	C224—C223—H223	119.6
C122—C123—H123	119.6	C222—C223—H223	119.6
C123—C124—C125	121.02 (19)	C223—C224—C225	121.15 (17)
C123—C124—H124	119.5	C223—C224—H224	119.4
C125—C124—H124	119.5	C225—C224—H224	119.4
C126—C125—C124	117.95 (18)	C224—C225—C226	118.11 (17)
C126—C125—C128	120.5 (2)	C224—C225—C228	120.54 (18)
C124—C125—C128	121.6 (2)	C226—C225—C228	121.34 (18)
C127—C126—C125	121.20 (18)	C227—C226—C225	121.02 (18)
C127—C126—H126	119.4	C227—C226—H226	119.5
C125—C126—H126	119.4	C225—C226—H226	119.5
C126—C127—C122	120.83 (18)	C226—C227—C222	120.50 (18)
C126—C127—H127	119.6	C226—C227—H227	119.7
C122—C127—H127	119.6	C222—C227—H227	119.7
C125—C128—H10J	109.5	C225—C228—H20J	109.5
C125—C128—H10K	109.5	C225—C228—H20K	109.5
H10J—C128—H10K	109.5	H20J—C228—H20K	109.5
C125—C128—H10L	109.5	C225—C228—H20L	109.5
H10J—C128—H10L	109.5	H20J—C228—H20L	109.5
H10K—C128—H10L	109.5	H20K—C228—H20L	109.5
C134—C129—C130	118.29 (16)	C230—C229—C234	118.28 (17)
C134—C129—P12	121.71 (14)	C230—C229—P22	119.14 (14)
C130—C129—P12	119.61 (13)	C234—C229—P22	122.39 (14)
C131—C130—C129	120.95 (17)	C231—C230—C229	120.67 (18)
C131—C130—H130	119.5	C231—C230—H230	119.7
C129—C130—H130	119.5	C229—C230—H230	119.7
C130—C131—C132	121.04 (17)	C232—C231—C230	121.23 (18)
C130—C131—H131	119.5	C232—C231—H231	119.4
C132—C131—H131	119.5	C230—C231—H231	119.4
C133—C132—C131	117.70 (17)	C231—C232—C233	118.07 (18)
C133—C132—C135	120.95 (17)	C231—C232—C235	121.01 (19)
C131—C132—C135	121.34 (18)	C233—C232—C235	120.89 (18)
C134—C133—C132	121.49 (17)	C234—C233—C232	121.15 (18)
C134—C133—H133	119.3	C234—C233—H233	119.4
C132—C133—H133	119.3	C232—C233—H233	119.4
C133—C134—C129	120.52 (17)	C233—C234—C229	120.60 (18)
C133—C134—H134	119.7	C233—C234—H234	119.7
C129—C134—H134	119.7	C229—C234—H234	119.7
C132—C135—H10M	109.5	C232—C235—H20M	109.5
C132—C135—H10N	109.5	C232—C235—H20N	109.5
H10M—C135—H10N	109.5	H20M—C235—H20N	109.5
C132—C135—H10O	109.5	C232—C235—H20O	109.5
H10M—C135—H10O	109.5	H20M—C235—H20O	109.5
H10N—C135—H10O	109.5	H20N—C235—H20O	109.5
C141—C136—C137	118.48 (16)	C237—C236—C241	118.21 (16)
C141—C136—P12	120.68 (13)	C237—C236—P22	119.27 (13)
C137—C136—P12	120.79 (14)	C241—C236—P22	121.57 (14)
C138—C137—C136	120.50 (18)	C238—C237—C236	121.00 (17)
C138—C137—H137	119.7	C238—C237—H237	119.5
C136—C137—H137	119.7	C236—C237—H237	119.5
C137—C138—C139	121.40 (18)	C237—C238—C239	120.98 (17)
C137—C138—H138	119.3	C237—C238—H238	119.5
C139—C138—H138	119.3	C239—C238—H238	119.5
C140—C139—C138	118.09 (17)	C240—C239—C238	117.90 (17)
C140—C139—C142	121.17 (19)	C240—C239—C242	121.04 (17)
C138—C139—C142	120.72 (18)	C238—C239—C242	121.04 (17)
C139—C140—C141	120.80 (18)	C239—C240—C241	121.39 (17)
C139—C140—H140	119.6	C239—C240—H240	119.3
C141—C140—H140	119.6	C241—C240—H240	119.3
C136—C141—C140	120.72 (17)	C240—C241—C236	120.48 (17)
C136—C141—H141	119.6	C240—C241—H241	119.8
C140—C141—H141	119.6	C236—C241—H241	119.8
C139—C142—H10P	109.5	C239—C242—H20P	109.5
C139—C142—H10Q	109.5	C239—C242—H20Q	109.5
H10P—C142—H10Q	109.5	H20P—C242—H20Q	109.5
C139—C142—H10R	109.5	C239—C242—H20R	109.5
H10P—C142—H10R	109.5	H20P—C242—H20R	109.5
H10Q—C142—H10R	109.5	H20Q—C242—H20R	109.5
			
N11—Fe1—P11—C108	−61.00 (9)	N22—Fe2—P21—C208	140.90 (8)
N12—Fe1—P11—C108	157.97 (9)	P22—Fe2—P21—C208	−103.03 (6)
P12—Fe1—P11—C108	52.26 (7)	N21—Fe2—P21—C201	130.16 (9)
N11—Fe1—P11—C115	175.76 (8)	N22—Fe2—P21—C201	−95.31 (9)
N12—Fe1—P11—C115	34.73 (9)	P22—Fe2—P21—C201	20.77 (7)
P12—Fe1—P11—C115	−70.98 (7)	N21—Fe2—P21—C215	−109.16 (8)
N11—Fe1—P11—C101	54.71 (8)	N22—Fe2—P21—C215	25.38 (8)
N12—Fe1—P11—C101	−86.32 (8)	P22—Fe2—P21—C215	141.45 (6)
P12—Fe1—P11—C101	167.97 (6)	N22—Fe2—N21—O21	−53 (11)
N12—Fe1—N11—O11	130 (19)	P22—Fe2—N21—O21	−178 (100)
P12—Fe1—N11—O11	−112 (19)	P21—Fe2—N21—O21	72 (11)
C108—P11—C101—C102	136.85 (15)	C208—P21—C201—C206	−5.77 (17)
C115—P11—C101—C102	−115.49 (15)	C215—P21—C201—C206	105.11 (16)
Fe1—P11—C101—C102	12.83 (16)	Fe2—P21—C201—C206	−133.75 (14)
C108—P11—C101—C106	−42.70 (16)	C208—P21—C201—C202	170.03 (14)
C115—P11—C101—C106	64.96 (16)	C215—P21—C201—C202	−79.08 (15)
Fe1—P11—C101—C106	−166.71 (13)	Fe2—P21—C201—C202	42.06 (16)
C106—C101—C102—C103	0.5 (3)	C206—C201—C202—C203	−0.1 (3)
P11—C101—C102—C103	−179.03 (15)	P21—C201—C202—C203	−176.12 (14)
C101—C102—C103—C104	−0.9 (3)	C201—C202—C203—C204	−0.3 (3)
C102—C103—C104—C105	0.1 (3)	C202—C203—C204—C205	0.5 (3)
C102—C103—C104—C107	179.78 (18)	C202—C203—C204—C207	−179.12 (18)
C103—C104—C105—C106	0.9 (3)	C203—C204—C205—C206	−0.3 (3)
C107—C104—C105—C106	−178.73 (18)	C207—C204—C205—C206	179.30 (19)
C104—C105—C106—C101	−1.3 (3)	C204—C205—C206—C201	−0.1 (3)
C102—C101—C106—C105	0.5 (3)	C202—C201—C206—C205	0.3 (3)
P11—C101—C106—C105	−179.94 (14)	P21—C201—C206—C205	176.06 (15)
C115—P11—C108—C113	33.99 (17)	C201—P21—C208—C213	90.85 (16)
C101—P11—C108—C113	142.08 (15)	C215—P21—C208—C213	−19.25 (18)
Fe1—P11—C108—C113	−96.19 (15)	Fe2—P21—C208—C213	−134.73 (14)
C115—P11—C108—C109	−152.98 (14)	C201—P21—C208—C209	−87.29 (15)
C101—P11—C108—C109	−44.89 (16)	C215—P21—C208—C209	162.61 (14)
Fe1—P11—C108—C109	76.84 (15)	Fe2—P21—C208—C209	47.13 (15)
C113—C108—C109—C110	−0.4 (3)	C213—C208—C209—C210	0.3 (3)
P11—C108—C109—C110	−173.66 (15)	P21—C208—C209—C210	178.59 (14)
C108—C109—C110—C111	−0.2 (3)	C208—C209—C210—C211	0.5 (3)
C109—C110—C111—C112	0.8 (3)	C209—C210—C211—C212	−0.8 (3)
C109—C110—C111—C114	−179.0 (2)	C209—C210—C211—C214	179.14 (19)
C110—C111—C112—C113	−0.7 (3)	C210—C211—C212—C213	0.3 (3)
C114—C111—C112—C113	179.0 (2)	C214—C211—C212—C213	−179.66 (19)
C111—C112—C113—C108	0.1 (3)	C209—C208—C213—C212	−0.9 (3)
C109—C108—C113—C112	0.5 (3)	P21—C208—C213—C212	−178.98 (14)
P11—C108—C113—C112	173.58 (15)	C211—C212—C213—C208	0.6 (3)
C108—P11—C115—C120	−132.53 (14)	C208—P21—C215—C216	123.32 (16)
C101—P11—C115—C120	121.59 (15)	C201—P21—C215—C216	14.76 (18)
Fe1—P11—C115—C120	−3.49 (16)	Fe2—P21—C215—C216	−116.98 (16)
C108—P11—C115—C116	46.96 (17)	C208—P21—C215—C220	−65.58 (16)
C101—P11—C115—C116	−58.92 (17)	C201—P21—C215—C220	−174.14 (14)
Fe1—P11—C115—C116	176.00 (13)	Fe2—P21—C215—C220	54.13 (15)
C120—C115—C116—C117	−0.4 (3)	C220—C215—C216—C217	−0.8 (3)
P11—C115—C116—C117	−179.90 (15)	P21—C215—C216—C217	170.19 (15)
C115—C116—C117—C118	1.5 (3)	C215—C216—C217—C218	1.5 (3)
C116—C117—C118—C119	−1.4 (3)	C216—C217—C218—C219	−0.4 (3)
C116—C117—C118—C121	178.78 (19)	C216—C217—C218—C221	178.2 (2)
C117—C118—C119—C120	0.3 (3)	C217—C218—C219—C220	−1.3 (3)
C121—C118—C119—C120	−179.88 (18)	C221—C218—C219—C220	179.96 (19)
C118—C119—C120—C115	0.8 (3)	C218—C219—C220—C215	2.1 (3)
C116—C115—C120—C119	−0.7 (3)	C216—C215—C220—C219	−1.0 (3)
P11—C115—C120—C119	178.82 (14)	P21—C215—C220—C219	−172.70 (15)
N11—Fe1—P12—C129	163.18 (8)	N21—Fe2—P22—C236	−59.05 (8)
N12—Fe1—P12—C129	−59.73 (8)	N22—Fe2—P22—C236	166.94 (8)
P11—Fe1—P12—C129	55.19 (7)	P21—Fe2—P22—C236	50.36 (7)
N11—Fe1—P12—C136	37.38 (9)	N21—Fe2—P22—C229	174.68 (8)
N12—Fe1—P12—C136	174.47 (8)	N22—Fe2—P22—C229	40.67 (9)
P11—Fe1—P12—C136	−70.61 (7)	P21—Fe2—P22—C229	−75.91 (7)
N11—Fe1—P12—C122	−79.57 (9)	N21—Fe2—P22—C222	59.68 (8)
N12—Fe1—P12—C122	57.52 (8)	N22—Fe2—P22—C222	−74.34 (9)
P11—Fe1—P12—C122	172.44 (6)	P21—Fe2—P22—C222	169.09 (6)
N11—Fe1—N12—O12	97.0 (16)	N21—Fe2—N22—O22	−21.2 (15)
P12—Fe1—N12—O12	−25.7 (16)	P22—Fe2—N22—O22	101.2 (15)
P11—Fe1—N12—O12	−136.5 (16)	P21—Fe2—N22—O22	−144.4 (15)
C129—P12—C122—C123	166.66 (15)	C236—P22—C222—C223	149.46 (14)
C136—P12—C122—C123	−84.59 (16)	C229—P22—C222—C223	−101.29 (15)
Fe1—P12—C122—C123	42.60 (16)	Fe2—P22—C222—C223	26.06 (15)
C129—P12—C122—C127	−16.60 (17)	C236—P22—C222—C227	−37.58 (18)
C136—P12—C122—C127	92.15 (16)	C229—P22—C222—C227	71.67 (17)
Fe1—P12—C122—C127	−140.65 (14)	Fe2—P22—C222—C227	−160.98 (15)
C127—C122—C123—C124	−1.4 (3)	C227—C222—C223—C224	−1.2 (3)
P12—C122—C123—C124	175.54 (16)	P22—C222—C223—C224	172.13 (14)
C122—C123—C124—C125	1.6 (3)	C222—C223—C224—C225	0.0 (3)
C123—C124—C125—C126	−0.3 (3)	C223—C224—C225—C226	1.2 (3)
C123—C124—C125—C128	−179.0 (2)	C223—C224—C225—C228	−178.34 (18)
C124—C125—C126—C127	−1.3 (3)	C224—C225—C226—C227	−1.2 (3)
C128—C125—C126—C127	177.5 (2)	C228—C225—C226—C227	178.35 (19)
C125—C126—C127—C122	1.5 (3)	C225—C226—C227—C222	0.0 (3)
C123—C122—C127—C126	−0.2 (3)	C223—C222—C227—C226	1.2 (3)
P12—C122—C127—C126	−176.92 (14)	P22—C222—C227—C226	−171.66 (16)
C136—P12—C129—C134	27.42 (17)	C236—P22—C229—C230	−149.74 (14)
C122—P12—C129—C134	133.38 (15)	C222—P22—C229—C230	100.68 (15)
Fe1—P12—C129—C134	−105.20 (14)	Fe2—P22—C229—C230	−19.72 (17)
C136—P12—C129—C130	−159.89 (14)	C236—P22—C229—C234	35.53 (17)
C122—P12—C129—C130	−53.92 (16)	C222—P22—C229—C234	−74.06 (16)
Fe1—P12—C129—C130	67.50 (15)	Fe2—P22—C229—C234	165.54 (13)
C134—C129—C130—C131	−0.5 (3)	C234—C229—C230—C231	0.3 (3)
P12—C129—C130—C131	−173.45 (14)	P22—C229—C230—C231	−174.69 (14)
C129—C130—C131—C132	0.8 (3)	C229—C230—C231—C232	−0.2 (3)
C130—C131—C132—C133	−0.7 (3)	C230—C231—C232—C233	−0.3 (3)
C130—C131—C132—C135	178.10 (19)	C230—C231—C232—C235	177.86 (18)
C131—C132—C133—C134	0.2 (3)	C231—C232—C233—C234	0.6 (3)
C135—C132—C133—C134	−178.60 (19)	C235—C232—C233—C234	−177.55 (18)
C132—C133—C134—C129	0.1 (3)	C232—C233—C234—C229	−0.5 (3)
C130—C129—C134—C133	0.0 (3)	C230—C229—C234—C233	0.0 (3)
P12—C129—C134—C133	172.80 (14)	P22—C229—C234—C233	174.83 (14)
C129—P12—C136—C141	−128.97 (15)	C229—P22—C236—C237	−153.01 (14)
C122—P12—C136—C141	123.45 (15)	C222—P22—C236—C237	−47.90 (16)
Fe1—P12—C136—C141	1.49 (17)	Fe2—P22—C236—C237	73.31 (15)
C129—P12—C136—C137	53.68 (16)	C229—P22—C236—C241	38.41 (16)
C122—P12—C136—C137	−53.91 (16)	C222—P22—C236—C241	143.52 (15)
Fe1—P12—C136—C137	−175.87 (12)	Fe2—P22—C236—C241	−95.27 (14)
C141—C136—C137—C138	0.0 (3)	C241—C236—C237—C238	−1.0 (3)
P12—C136—C137—C138	177.39 (14)	P22—C236—C237—C238	−169.96 (14)
C136—C137—C138—C139	−0.1 (3)	C236—C237—C238—C239	−0.9 (3)
C137—C138—C139—C140	0.4 (3)	C237—C238—C239—C240	2.1 (3)
C137—C138—C139—C142	−177.94 (18)	C237—C238—C239—C242	−176.25 (17)
C138—C139—C140—C141	−0.5 (3)	C238—C239—C240—C241	−1.4 (3)
C142—C139—C140—C141	177.75 (18)	C242—C239—C240—C241	176.92 (16)
C137—C136—C141—C140	−0.2 (3)	C239—C240—C241—C236	−0.5 (3)
P12—C136—C141—C140	−177.58 (14)	C237—C236—C241—C240	1.7 (3)
C139—C140—C141—C136	0.4 (3)	P22—C236—C241—C240	170.36 (13)
N21—Fe2—P21—C208	6.36 (8)		
